# Clinical indications for host-cell DNA methylation markers in cervical screening and management of cervical intraepithelial neoplasia: A review

**DOI:** 10.1016/j.tvr.2024.200308

**Published:** 2024-12-16

**Authors:** S. Dick, D.A.M. Heideman, J. Berkhof, R.D.M. Steenbergen, M.C.G. Bleeker

**Affiliations:** a Amsterdam UMC, Location Vrije Universiteit Amsterdam, Pathology, Boelelaan, 1117, Amsterdam, the Netherlands; b Cancer Center Amsterdam, Imaging and Biomarkers, Amsterdam, the Netherlands; c Amsterdam UMC, Location Vrije Universiteit Amsterdam, Epidemiology and Data Science, Amsterdam, the Netherlands

**Keywords:** DNA methylation, Cervical cancer, Cervical screening, Human papillomavirus, Cervical intraepithelial neoplasia, Triage test, Conservative treatment, Management of CIN, Post-treatment monitoring, Host-cell DNA methylation

## Abstract

DNA methylation of host-cell genes is an epigenetic process that regulates gene expression and is associated with cervical cancer development. Studies on the natural history of cervical intraepithelial neoplasia (CIN) and the molecular alterations associated with cervical carcinogenesis led to the identification of several host-cell DNA methylation markers. Over the past years, various studies on methylation markers have shown promising results in terms of diagnostic and prognostic value to improve cervical cancer screening and management of CIN. This review provides an overview of the clinical indications of host-cell DNA methylation markers in cervical screening and management of CIN, and outlines avenues for further applications.

## Highlights

• Methylation of host-cell genes is an epigenetic process that regulates gene expression.
• Methylation markers have demonstrated value in diagnosis, treatment, and post-treatment monitoring.
• DNA methylation consistently identified CIN2/3 lesions with highest risk of progression to cancer.
• DNA methylation markers can help to further optimize screening and management.

## 1 Introduction

Cervical screening programs aim to detect and subsequently treat cervical intraepithelial neoplasia (CIN) lesions in order to prevent progression to cervical cancer. Primary human papillomavirus (HPV)-based testing is currently preferred in cervical screening programs, because of its very high sensitivity for CIN3 and cancer [1,2]. However, most HPV infections are transient and additional triage testing is required to reduce unnecessary colposcopy referrals of HPV-positive women without clinically relevant cervical disease. At present, cytology is the most frequently used triage test, but has limitations in terms of sensitivity, subjectivity and applicability, and a repeat test is needed to ensure sufficient protection [3,4]. Another disadvantage of HPV and/or cytology testing is that it insufficiently reflects the stage of underlying cervical disease and in case of CIN2/3 the risk of progression to cervical cancer. Studies on the natural history of CIN and the molecular alterations associated with cervical carcinogenesis led to the identification of host-cell DNA methylation markers. These markers reflect the carcinogenic process and therefore offer the opportunity to improve cervical screening and management of women with CIN to prevent overreferral and overtreatment. This review gives an overview on the potential clinical indications of host-cell DNA methylation markers in cervical screening and management of CIN and outlines possibilities for future applications. We focused on studies evaluating host-cell DNA methylation in clinician-collected cervical samples for the detection of CIN3+ and management of CIN2/3.

## 2 Methylation markers in cervical carcinogenesis

DNA hypermethylation of promoter regions of tumour suppressor genes is an epigenetic process that silences gene expression. This process is, in part, induced by the HPV E7 oncoprotein and represents an early event contributing to cervical cancer development [[5], [6], [7], [8]]. Several methylation markers for the detection of CIN3 and cervical cancer have been identified using targeted and genome-wide approaches including *CADM1* [9]*, MAL* [10]*, FAM19A4* [11]*, miR124-2* [12], *DLX1* [13]*, ITG4* [13]*, RXFP3* [13]*, SOX17* [13]*, ZNF671* [13], PAX1 [14], *SOX1* [14,15]*, ASCL1* [16]*, LHX8* [16]*, ST6GALNAC5* [16], *GHSR* [17], *SST* [17], *ZIC1* [17]*, JAM3* [18], *EPB41L3* [18]*, C13orf18* [19]*,* ANKRD18CP [20], *ZSCAN1* [20]*, DPP6* [21], *RALYL* [21], *GSX1* [21]*, ATP10A* [22], *HAS1* [22], *POU4F3* [23] *ZNF582* [24] and *TRH* [25]. A few methylation markers or marker panels are commercially available: the QIAsure Methylation Test targeting *FAM19A4/miR124-2* (Qiagen, Hilden, Germany), the GynTect assay targeting *ASTN1/DLX1/ITGA4/RXFP3/SOX17/ZNF671* (Oncgnostics, Jena, Germany), the CONFIDENCE Marker assay targeting *POU4F3* (NEUMANN Diagnostics Ltd., Budapest, Hungary) and the Cervi-M test targeting *PAX1* (iStat Biomedical Co. Ltd., New Taipei City, Taiwan).

### 2.1 Detection of cervical cancer and CIN

DNA methylation markers offer a high sensitivity for cervical cancer. In a large series of 519 cervical cancers, the *FAM19A4/miR124-2* methylation test showed a positivity rate of 98.3 % [26]. Cancer detection was independent of HPV status, histotype, FIGO stage, geographical region or sample type. Smaller series of cervical cancers evaluated for *FAM19A4*, *PAX1*/*ZNF582/SOX1*, *CADM1/MAL/miR124-2*, *ASTN1/DLX1/ITGA4/RXFP3/SOX17/ZNF671*, *ANKRD18CP/C13ORF18/EPB41L3/JAM3/SOX1/ZSCAN1, ASCL1/LHX8/ST6GALNAC5, GHSR/SST/ZIC1*, *ZNF582, PAX1* and *RARB* showed comparable results with cancer positivity rates between 93 % and 100 % [[27], [28], [29], [30], [31], [32], [33], [34], [35], [36], [37], [38], [39], [40], [41], [42], [43], [44], [45], [46]].

Interestingly, methylation patterns of high-grade CIN (i.e., CIN2/3) showed substantial heterogeneity. In a study evaluating 12 methylation markers, three-quarters of CIN3 and half of CIN2 had a cancer-like methylation pattern [47]. It was found that CIN2/3 lesions associated with a long-term (≥5 years) HPV infection (i.e., advanced lesions) had significantly higher methylation levels compared with CIN2/3 lesions with a more recently acquired (<5 years) HPV infection (i.e., early or incident lesions) [17,48,49]. These findings suggest that methylation-positive CIN2/3 lesions may have a high risk of progression to cancer. Methylation markers evaluated in HPV-positive-women <30 years and ≥30 years showed that women <30 years with CIN2/3 had lower methylation positivity rates compared with women ≥30 years [13,21,38,[50], [51], [52], [53]]. This lower positivity rate is probably related with the shorter duration of the associated HPV infection and a likely lower risk of progression to cancer in these women. Regression rates in young women have also shown to be very high with estimates up to 66 % [54,55]. The lower methylation positivity rates in women <30 years likely reflect the association between cancer risk and the duration since onset of CIN2/3. Importantly, the methylation status detected in clinician-collected cervical samples has been shown to correspond with the most severe lesion in case of multiple lesions [56]. To summarize, methylation analysis in clinician-collected cervical samples enables the detection of cervical lesions in need of treatment.

## 3 Methylation markers in cervical screening

### 3.1 Triage of HPV-positive women

Methylation markers have been evaluated in many studies as an alternative to cytology triage in HPV-based cervical screening. Important requirements for a triage strategy are [1] a high cross-sectional sensitivity for CIN3 and cervical cancer to ensure safety [2], a high specificity to keep unnecessary colposcopy referrals low and [3] a high long-term performance to ensure safety of triage-negative women after dismissal from surveillance [4]. Table 1 presents an overview of the current literature published until June 15, 2024 regarding the performance of methylation markers in the detection of CIN3+ in HPV-positive women. The triage performance of the QIAsure Methylation Test (Qiagen) was assessed in a *post hoc* analysis and showed a similar sensitivity compared with cytology (71.3 % vs 76.0 %), but a lower specificity (78.3 % vs 87.0 %) [41]. Comparable results of the QIAsure Methylation Test were reported in a large European multicentre study, with a CIN3 sensitivity of 77.2 % and a specificity of 78.3 % [42]. *FAM19A4* and/or *miR124-2* methylation were evaluated in several other studies reporting similar results with sensitivities of 67 %–78 % and specificities of 61 %–91 % [28,[57], [58], [59], [60]]. The GynTect assay (Oncgnostics), as evaluated in two studies showed a sensitivity of 67.4 % and 66.7 %, and a specificity of 76.0 % and 84.1 % for the detection of CIN3+, respectively [36,57]. The CONFIDENCE Marker test (NEUMANN Diagnostics Ltd.) showed a sensitivity of 72.3 % and a specificity of 76.5 % for CIN3+ detection [61]. In a training and validation study, combined *ASCL1/LHX8* methylation showed a cross-validated CIN3 sensitivity of 83.4 % and a specificity of 82.4 % [30]. Validation of this bi-marker panel in an independent cohort from a Dutch cervical screening study resulted in a CIN3+ sensitivity of 76.9 % and a specificity of 74.5 % in HPV-positive women [62]. The somewhat lower performance may be explained by the inclusion of women with CIN2 and the fact that the samples were derived from primary HPV-based screening whereas the previous study used a case-control setting.

**Table 1:** Overview of the performance of host-cell DNA methylation markers as a triage test on clinician-collected cervical samples in HPV-positive women.

Study	Year of publication	Country	Study population	Number of women	Commercial test	Methylation marker(s)	Sensitivity (%)	Specificity (%)	PPV (%)	NPV (%)	Endpoint
Vink et al. [41]	2021	The Netherlands	Screening	979	QIAsure	*FAM19A4/miR124-2*	71.3	78.3	33.0	94.8	CIN3+
Bonde et al. [44]	2021	Europe	Screening	2384	QIAsure	*FAM19A4/miR124-2*	77.2	78.3	28.3	96.9	CIN3
Bu et al. [28]	2018	China	Selection of HPV + samples	215	N/A	*FAM19A4*	75.4	81.4	–	–	CIN3+
Dippmann et al. [57]	2020	Germany	Referral population	149	QIAsure	*FAM19A4/miR124-2*	78.6	68.2	–	–	CIN3+
Kaliff et al. [58]	2022	Sweden	Screening	476	QIAsure	*FAM19A4/miR124-2*	67.2	27.7	–	–	CIN3+
Al Roomy et al. [59]	2023	Kuwait	Selection of HPV + samples	25	QIAsure	*FAM19A4/miR124-2*	75	94.4	–	–	CIN3
Dankai et al. [60]	2019	Thailand	Screening	115	N/A	*FAM19A4*	68.2	60.6	75.4	63.5	CIN3+
Schmitz et al. [36]	2017	Germany	Referral population	306	Gyntect	*ASTN1/DLX1/ITGA4/RXFP3/SOX17/ZNF671*	67.4	76	–	–	CIN3+
Dippmann et al. [57]	2020	Germany	Referral population	149	Gyntect	*ASTN1/DLX1/ITGA4/RXFP3/SOX17/ZNF671*	66.7	84.1	–	–	CIN3+
Kocsis et al. [61]	2017	Hungary	Screening and referral	1287	CONFIDENCE	*POU4F3*	72.3	76.5	–	–	CIN3+
Dick et al. [30]	2020	The Netherlands	Selection of HPV + samples	527	N/A	*ASCL1/LHX8*	83.4	82.4	–	–	CIN3
Verhoef et al. [62]	2022	The Netherlands	Screening	715	N/A	*ASCL1/LHX8*	76.9	74.5	30.6	95.7	CIN3+
Dankai et al. [60]	2019	Thailand	Screening	115	N/A	*CADM1*	70.5	60.6	52.5	76.8	CIN3+
Dankai et al. [60]	2019	Thailand	Screening	115	N/A	*MAL*	72.7	50.7	47.8	75	CIN3+
Verhoef et al. [37]	2015	The Netherlands	Screening non-attendees	364	N/A	*CADM1/MAL*	69.4	71.2	33.1	91.9	CIN3+
Overmeer et al. [63]	2011	The Netherlands	Screening	64	N/A	*CADM1/MAL*	70	78	–	–	CIN3+
De Strooper et al. [64]	2014	The Netherlands	Screening	250	N/A	*CADM1/MAL*	68.4	75.5	35.1	92.5	CIN3+
Hesselink et al. [65]	2011	The Netherlands	Screening	236	N/A	*CADM1/MAL*	68.4	75.3	34.7	92.5	CIN3+
Tian et al. [39]	2017	China	Screening	312	N/A	*ZNF582*	77.4	77.7	–	–	CIN3+
Tian et al. [39]	2017	China	Screening	312	N/A	*ZNF582/PAX1*	86.5	65	–	–	CIN3+
Li et al. [66]	2019	China	Meta-analysis	1749	N/A	*ZNF582*	75	87	–	–	CIN3+
Su et al. [67]	2022	China	Unknown	424	N/A	*ZNF582*	83.9	93.1	–	–	CIN3+
Zhu et al. [69]	2023	China	Referral	391	N/A	*ZNF671*	78.5	67.4	62.0	82.2	CIN3+
Yang et al. [70]	2023	China	Referral	381	N/A	*PAX1*	90	69.3	14.0	99.2	CIN3+
Su et al. [67]	2022	China	Unknown	424	N/A	*PAX1*	77.4	90.6	–	–	CIN3+
Chang et al. [68]	2021	Taiwan	Screening	470	N/A	*PAX1*	76.9	91.6	45.5	97.8	CIN3+
Van Leeuwen et al. [71]^a^	2019	Slovenia	Screening	235	N/A	*ANKRD18CP/C13orf18/JAM3*	68.4	66.7	15.3	96	CIN3+
Li et al. [44]	2021	China	Screening and referral	227	N/A	*ANKRD18CP/C13orf18/JAM3*	90	63	–	–	CIN3+
Van Leeuwen et al. [71]^a^	2019	Slovenia	Screening	235	N/A	*C13ORF18/EPB41L3/JAM3*	94.7	63.9	18.8	99.3	CIN3+
Li et al. [44]	2021	China	Screening and referral	227	N/A	*C13ORF18/EPB41L3/JAM3*	93	58	–	–	CIN3+
Van Leeuwen et al. [71]^a^	2019	Slovenia	Screening	235	N/A	*SOX1/ZSCAN1*	78.9	81.5	27.3	97.8	CIN3+
Li et al. [44]	2021	China	Screening and referral	227	N/A	*SOX1/ZSCAN1*	89	65	–	–	CIN3+
Herzog et al. [53]	2022	Sweden	Screening and referral	506	N/A	*DPP6/RALYL/GSX1*	78	90	59.0	92	CIN3+
Schreiberhuber et al. [72]^a^	2024	Sweden	Screening	2138	N/A	*DPP6/RALYL/GSX1*	85.1	76.4	16.9	98.9	CIN3+

Abbreviations: CIN3+, cervical intraepithelial neoplasia grade 3 or worse; N/A, not applicable; NPV, negative predictive value; PPV, positive predictive value.

a Sensitivity, specificity, NPV and PPV were re-calculated using data from the original papers.

*CADM1/MAL* methylation in HPV-positive women showed sensitivities of 68.4–86.8 % with specificities of 50.7–78 % for CIN3+ detection [37,60,[63], [64], [65]]. *ZNF582, ZNF671* and *PAX1* showed for the triage of HPV-positive women sensitivities between 75 % and 90 % and specificities between 65 % and 93.1 % for the detection of CIN3+ [39,[66], [67], [68], [69], [70]]. *ANKRD18CP/C13orf18/JAM3*, *C13ORF18/EPB41L3/JAM3 and SOX1/ZSCAN1* were evaluated as a triage test in a Slovenian and Chinese population and showed sensitivities between 68.4 % and 93 % and specificities between 58 % and 81.5 % [43,71].

A recently developed methylation marker panel, the WID-qCIN including *DPP6, RALYL,* and *GSX1,* showed a sensitivity of 78 % for the detection of CIN3+. The specificity was 90 %, but also some HPV-negative controls were included [53]. The WID-qCIN assay was then optimized and transformed from a singleplex-to a duplex-based reaction and evaluated in a cohort of HPV-positive women ≥30 years from the cervical screening program in Sweden [72]. The optimized WID-qCIN test showed a sensitivity of 85.1 % and a specificity of 76.4 % for the detection of prevalent CIN3+.

Of note, proper comparison of methylation results is hampered by the absence of a pre-defined threshold for methylation positivity in most studies using a non-commercial research test and the analysis of both screening and referral populations which have different case control ratios.

A meta-analysis of various methylation markers with studies included until December 2018 reported a sensitivity for CIN3+ detection of 70.5 % (95 % CI: 64.8–75.6) with a specificity of 74.5 % (95 % CI: 70.8–78.1) [73]. In this pooled analysis, DNA methylation testing had a similar CIN3+ sensitivity and a higher specificity compared with cytology at a cut-off of ≥ASC-US. Compared with HPV16/18 genotyping, DNA methylation showed a similar CIN3+ sensitivity and specificity. In this meta-analysis, most, but not all studies, included HPV-positive women. In a more recent meta-analysis, DNA methylation was evaluated as a triage test in HPV-positive women with studies included until April 2021. It showed a pooled sensitivity of 81 % (95 % CI: 76–85) with a pooled specificity of 75 % (95 % CI: 70–79) for CIN3+ detection in clinician-collected cervical samples [74]. Methylation markers showed a higher specificity with an equivalent sensitivity compared with cytology. However, one of the major limitations of these meta-analysis is that studies evaluating DNA methylation markers use different methodologies and protocols.

Long-term performance of methylation markers in HPV-positive women is important to determine the safety of surveillance schemes. The *FAM19A4/miR124-2* methylation test is the only test of which long-term longitudinal data are available. In two Dutch screening cohorts, the 14-year cervical cancer and CIN3+ risk in HPV-positive women was evaluated after a negative methylation result and compared with cytology [40,41,75]. In the POBASCAM study, the 14-year cervical cancer risk was significantly lower in methylation-negative women (1.7 %) than in cytology-negative women (2.4 %) [40]. The 14-year CIN3+ risk was equal after a negative methylation (16.3 %) or a negative cytology test (15.6 %) [75]. In the VUSA-Screen study, the 14-year cervical cancer risk was significantly lower in methylation-negative women (1.1 %) compared with cytology-negative women (2.0 %). The 14-year CIN3+ risk was equal after a negative methylation (12.8 %) and a negative cytology test (12.8 %) [41]. These results demonstrated that methylation markers provided an equal or better long-term safety compared with cytology and therefore methylation triage could be a safe alternative to cytology triage.

The potential of methylation to predict CIN3+ in HPV-positive women with normal cytology in the near future was evaluated using the WID-CIN test and WID-qCIN test, showing that future CIN3+ (after 1–4 years) were detected at 55 % sensitivity and 75 % specificity and 52 % sensitivity and 94 % specificity, respectively [21,53]. Of note, sensitivity decreased with increasing time interval. *FAM19A4/miR124-2* was also evaluated in cytology samples 4 months to 8 years before histological diagnosis of CIN3+ [76]. Methylation was significantly more present in women who developed CIN3+ compared with women without CIN3+ in follow-up. Methylation was also more often positive compared with cytology at a cut-off of ≥ASC-US prior to CIN3+ diagnosis.

Considering the costs of DNA methylation as a triage test in cervical screening, it was modelled that the QIAsure Methylation Test targeting *FAM19A4/miR124-2* reduced the cost per screen compared with cytology triage and fewer people were lost to follow-up [77].

### 3.2 Triage of HPV-positive women with ASC-US/LSIL

Since the implementation of HPV-based screening with cytology triage, there is a marked increase in the colposcopy referral rate and only a moderate increase in the detection of CIN2/3 and cervical cancer [[78], [79], [80], [81], [82]]. In the Netherlands, the colposcopy referral rate showed a two-fold increase [[83], [84], [85]], which is mainly caused by the direct referral of HPV-positive women with ASC-US/LSIL. In approximately 60 % of these women referred for colposcopy, no clinically relevant disease (≤CIN1) was detected [85].

Multiple studies have evaluated DNA methylation as an additional triage test in HPV-positive women with ASC-US/LSIL to reduce the colposcopy referral rate. In a *post hoc* analysis of two Dutch screening studies, the *FAM19A4/miR124-2* methylation test showed that the number of direct colposcopy referrals can be reduced in HPV-positive women with ASC-US/LSIL while maintaining clinical CIN3+ sensitivity [86]. The CIN3+ risk after a positive methylation result was 33.1 % compared with 9.8 % after a negative methylation result. This strategy could reduce colposcopy referrals with 59 %. In comparison, HPV16/18 genotyping as an additional triage test in HPV-positive women with ASC-US/LSIL resulted in a 27.6 % CIN3+ risk in the HPV16/18-positive group and a 13.2 % CIN3+ risk in the HPV16/18-negative group. This strategy would reduce colposcopy referrals with 57 %. An accepted threshold for dismissal from further follow-up is a minimal CIN3+ NPV of 98 %, which translates into a maximum CIN3+ risk of 2 % after a negative test [4]. For both *FAM19A4/miR124-2* methylation and HPV16/18 genotyping, the CIN3+ risk after a negative test was too high (10 % and 13 %, respectively) to recommend return to the regular screening program. By combining DNA methylation with HPV16/18/31/33/45 extended genotyping, the CIN3+ risk after a negative test decreased to 2.8 %. This strategy would reduce the colposcopy referral rate with 25 %.

Similar results on triage of HPV-positive women with ASC-US/LSIL were observed in a large European multicentre study reporting a 66 % reduction in colposcopy referrals after stratification with *FAM19A4/miR124-2* methylation [42]. The best discriminatory value of *FAM19A4/miR124-2* was achieved in HPV-positive women with ASC-US/LSIL compared with other cytological results. In another study, *ASCL1/LHX8* methylation showed a CIN3+ risk of 22.6 % after a positive methylation test and 5.2 % after a negative test, resulting in a 70 % reduction in colposcopy referrals [62].

Since the a priori risk of CIN3+ in women with ASC-US/LSIL is low, DNA methylation can be used to decide between immediate referral for colposcopy or follow-up after 6–12 months.

### 3.3 Screening in WLHIV

Studies on DNA methylation in cervical screening in low and middle income countries (LMIC) and particularly women living with HIV (WLHIV) are summarized in Table 2. *CADM1/MAL/miR124-2* methylation for the *triage* of HPV-positive women in a South African screening cohort of WLHIV and in a Kenyan cohort, yielded CIN3+ sensitivities of 73.8 % and 79 % with specificities of 81.5 % and 70 %, respectively [87,88]. *FAM19A4/miR124-2* methylation also showed a high sensitivity of 72.9 % and a specificity of 76.1 % as a triage test in the same South African screening cohort of WLHIV [89]. Similar results were found for *EPB41L3* methylation in WLHIV from South Africa and Burkina Faso, showing a CIN2+ sensitivity of 60.7 % and a specificity of 69.7 % [90].

**Table 2:** Overview of the performance of host-cell DNA methylation markers in WLHIV.

Study	Year of publication	Country	Primary screening or triage test	Study population	Number of women	Commercial test	Methylation marker(s)	Sensitivity (%)	Specificity (%)	Endpoint
Van Zummeren et al. [87]	2017	South Africa	Triage	Cohort of WLHIV	321	N/A	*CADM1/MAL/miR124-2*	73.8	81.5	CIN3+
De Vuyst et al. [88]	2015	Kenya	Triage	Cohort of WLHIV	248	N/A	*CADM1/MAL/miR124-2*	79	70	CIN3+
Kremer et al. [89]	2019	South Africa	Triage	Cohort of WLHIV	285	QIAsure	*FAM19A4/miR124-2*	72.9	76.1	CIN3+
Kelly et al. [90]	2018	South Africa/Burkina Faso	Primary	Selection of samples	268	N/A	*EPB41L3*	69.7	66.2	CIN2+
Kremer et al. [91]	2018	South Africa	Primary	Cohort of WLHIV and referral population	429	N/A	*ASCL1*	72.1	75	CIN3+
Kremer et al. [91]	2018	South Africa	Primary	Cohort of WLHIV and referral population	429	N/A	*LHX8*	73.8	75	CIN3+
Kremer et al. [91]	2018	South Africa	Primary	Cohort of WLHIV and referral population	429	N/A	*ST6GALNAC5*	55.7	75	CIN3+
Vink et al. [92]	2023	South Africa	Primary	Cohort of WLHIV	411	N/A	*ASCL1/LHX8*	84.6	86.7	CIN3+

Abbreviations: CIN3+, cervical intraepithelial neoplasia grade 3 or worse; N/A, not applicable; WLHIV, women living with HIV.

*ASCL1, LHX8* and *ST6GALNAC5* were evaluated as a *primary* screening test and showed a CIN3+ sensitivity of 72.1 %, 73.8 % and 55.7 %, respectively, at a predefined specificity of 75 % in a WLHIV South African cohort [91]. Validation of combined *ASCL1*/*LHX8* markers in an independent South African cohort of WLHIV demonstrated a sensitivity of 84.6 % and a specificity of 86.7 % for CIN3+ detection [92]**.** Hence, DNA methylation could provide a useful alternative to primary cytology or primary HPV screening.

## 4 Methylation markers for clinical management of CIN2/3

Clinical management of women referred for colposcopy depends largely on the histology result of colposcopy-guided biopsies. However, it remains a challenge to morphologically distinguish regressive from progressive CIN2/3 lesions. Currently, women with CIN3 and the majority of CIN2 are treated, but guidelines vary between clinics and countries. Overtreatment is specifically critical among women of reproductive age and pregnant women, as it may lead to adverse pregnancy outcomes [93,94]. Many countries have changed their guidelines in recent years in which a wait-and-see policy is advised in women of reproductive age with CIN2 [95].

Accurate and consistent histological grading of CIN2/3 lesions is therefore important to decide between conservative management and excisional treatment. Histological grading of CIN is only moderately reproducible and, given the morphologic spectrum, borders between CIN grades are difficult to define [[96], [97], [98], [99], [100]]. Especially the diagnosis of CIN2 is challenging, as it constitutes of a heterogeneous group that can be the result of a productive HPV infection with a high chance of regression or a transforming HPV infection with a high chance of progression to cervical cancer [101,102].

### 4.1 Clinical management of CIN2/3

The *FAM19A4/miR124-2* methylation test was evaluated in the CONCERVE study, an observational clinical trial, to predict clinical regression of CIN2/3 [103]. One hundred twelve women aged 20–53 years with histologically-confirmed CIN2/3 were prospectively followed for 24 months. Women with a negative *FAM19A4/miR124-2* methylation result on the baseline clinician-collected sample showed more clinical regression (74.7 %) than women with a positive methylation result (51.4 %, *p* = 0.013). Of interest, the association between clinical regression and methylation was similar on clinician-collected and self-collected samples. Women with a negative methylation result combined with ASC-US/LSIL (88.4 %) or a HPV16-negative result (85.1 %) showed the highest regression rates. In another study, *EPB41L3* was evaluated as a prognostic biomarker to identify regressive or progressive CIN2 in 149 women aged <27 years [104]. Methylation levels of women in the progression group were significantly higher compared with the regression and persistence group. DNA methylation of *ASTN1/DLX1/ITGA4/RXFP/SOX17/ZNF671* was evaluated in 50 women aged 18–29 years with histologically-confirmed CIN2 or CIN3 as a predictor of lesion regression in the GynTect-Pro study [105]. Women were prospectively monitored every 6 months for a period of 12 months (CIN3) or 24 months (CIN2). Women with a negative methylation test showed regression in 67 % in case of CIN2 and 56 % in case of CIN3. These data underline the value of methylation analysis to provide guidance on treatment and to justify a wait-and-see policy in women with CIN2/3. Several studies are ongoing to assess the value of methylation to predict the biological behaviour of CIN2/3 lesions [106].

### 4.2 Post-treatment monitoring of CIN2/3

Treatment of CIN2/3 by Large Loop Excision of the Transformation Zone (LLETZ) or conization can prevent progression to cancer. After treatment prolonged follow-up is needed because these women remain at increased risk of developing recurrent CIN or cervical cancer [[107], [108], [109], [110]]. Recurrent CIN include both residual lesions, resulting from an incomplete excision, and newly developed lesions. Differentiation between residual and newly developed CIN2/3 is relevant because of the presumed difference in short-term risk of progression to cervical cancer. Guidelines for post-treatment monitoring should ideally be based on biomarkers that can differentiate between residual lesions that require re-treatment, and newly developed CIN with a low short-term risk of progression to cancer which can be managed conservatively.

*CADM1/MAL* methylation analysis in a multicentre prospective trial among 364 women treated for CIN2/3 showed that 64 % of recurrent CIN3 and 17–22 % of ≤CIN1 tested methylation positive in clinician-collected cervical samples [111]. In a *post hoc* analysis of the same study, methylation marker panels *ASCL1/LHX8* and *FAM19A4/miR124-2* showed an improved CIN3 detection rate of >92.9 %, while detection rate of ≤CIN1 was <8.9 % [112]. In this study, DNA methylation testing showed a similar CIN3 sensitivity compared with HPV testing and cytology, while specificity was significantly better. An important finding was that recurrent CIN2/3 caused by a persistent HPV infection had significantly higher methylation positivity rates compared with recurrent CIN2/3 and an incident or absent HPV infection. In WLHIV from South-Africa, *ASCL1/LHX8* showed a 4-year recurrent CIN2/3 risk after a negative methylation test similarly low as compared with a negative cytology result or a negative HPV result [113]. In conclusion, methylation analysis provides a good alternative to HPV and/or cytology post-treatment monitoring enabling the differentiation between re-treatment and conservative management in case of recurrent CIN. Implementation awaits further studies assessing the CIN2/3 risk after a negative test result to determine follow-up intervals after treatment and ensure safety.

## 5 Future applications

### 5.1 Other clinical indications

Since information on DNA methylation status reflects the severity and progressive potential of the underlying cervical disease, methylation assays may also be used for other indications. In studies evaluating the long-term performance of DNA methylation analysis, it was shown that HPV-positive methylation-negative women had a lower 14-year cervical cancer risk compared with HPV-positive cytology-negative women [40,41]. A negative methylation test for HPV-positive women 60–65 years may possibly reassure these women against cervical cancer when leaving the cervical screening program. In a study on *PAX1* methylation in HPV-positive women aged ≥65 years, methylation showed a better performance compared with cytology in the detection of CIN3+ [68]. However, in a pilot study evaluating *FAM19A4/miR124-2* DNA methylation among 48 women 55–59 years who exited the cervical screening program, no benefit of DNA methylation was found compared with cytology triage [114]. Since HPV-negative cervical cancer and other HPV-independent gynaecological tumours are more common at older age, it may also be interesting to study methylation testing as a primary test or co-test in the last round of screening [115]. DNA methylation on clinician-collected cervical samples has shown that it can also be evaluated for the presence of endometrial cancer [116,117]. The lower sensitivity of cytology testing at older age caused by atrophic changes in postmenopausal women and a transformation zone situated higher in the cervical canal [[118], [119], [120]], also advocates for DNA methylation testing at the final screening round. When monitoring patients after cervical cancer treatment, there is sometimes uncertainty about the significance of cytological abnormalities, either with or without persistent HPV detection. Uncertainty can also arise in other cases with discrepancies between cytological findings, HPV result and colposcopic impression. In such cases, a methylation test can provide additional objective information. A recent study on pregnant women with CIN3 showed that DNA methylation could help to rule out progressive CIN to justify conservative treatment until after delivery [121]. The entrance of HPV-vaccinated women in cervical screening asks for adaptations since the CIN3+ risk is strongly reduced [[122], [123], [124]]. HPV-vaccinated women will likely have less clinically relevant lesions, since these lesions are mainly caused by non-vaccine targeted HPV genotypes, which are low oncogenic HPV genotypes. In a first study evaluating methylation levels in HPV-vaccinated women no significant differences in methylation levels were found between HSIL, LSIL and controls indicating that most cervical lesions among HPV-vaccinated women could regress without treatment [125]. More systematic studies are required to confirm these extended indications.

### 5.2 Self-sampling

The implementation of self-sampling to attract underscreened women and non-attendees into screening has been adopted in many countries over the past years [126]. Self-sampling lowers the barrier to screening and is widely accepted by women, as a result of which self-collected samples are increasingly used in cervical screening programs [127]. The clinical accuracy of PCR-based HPV testing on a self-collected sample for detection of CIN3+ is high and supports use of HPV self-sampling as a primary screening test for all women [128]. While cytology cannot be accurately performed on self-collected samples, evaluations of methylation markers as triage test on HPV-positive self-collected samples have demonstrated good clinical performance for CIN3+ [16,[129], [130], [131], [132], [133], [134], [135]]. First evaluations of methylation markers in urine for CIN3 and cervical cancer detection also revealed promising results [[136], [137], [138]]. Self-collected samples provide another sample type to be used for methylation analysis in any of the clinical indications as discussed in this review.

## 6 Implementation in cervical screening programs and clinical practice

Implementation of methylation markers in cervical screening programs would be most beneficial for women with ASC-US/LSIL to reduce the number of direct referrals and overtreatment of ≤CIN1. However, direct comparison of studies evaluating methylation markers is difficult because of different techniques, indications, endpoints, and populations. Ideally, prospective and longitudinal studies within screening programs should be conducted using and comparing different methylation tests. Preferably, these performance studies include tests with predefined thresholds for which robustness and reproducibility has been demonstrated, and that meet regulatory requirements such as CE-IVDR marking. An advantage of DNA methylation is that positivity in cervical cancers has been shown to be independent of HPV genotype, so DNA methylation will also detect cancers attributed to HPV types that are not targeted by the HPV vaccines [26]. Further research is ongoing to evaluate DNA methylation in HPV-vaccinated cohorts [125,139]. Recently, the application of methylation testing in clinical practice to guide decision between conservative management and excisional treatment of CIN2/3 has started in the Netherlands. We expect that DNA methylation tests will become increasingly available in other countries.

## 7 Conclusions

Studies on methylation markers have confirmed the potential fields of application including diagnosis, management, and post-treatment surveillance of CIN2/3. Methylation markers provide a valuable triage method for HPV-positive women in cervical cancer screening with several advantages over traditional methods. DNA methylation identifies CIN2/3 lesions at highest risk of progression to cervical cancer and methylation analysis has shown a high level of accuracy and reproducibility [140]. Cytology, in contrast, relies on subjective interpretation, and is more prone to training and interpretational errors. DNA methylation markers can help to further optimize screening and management and reduce referral and overtreatment of women without clinically relevant disease.

## CRediT authorship contribution statement

**S. Dick:** Writing – review & editing, Writing – original draft, Methodology, Data curation, Conceptualization. **D.A.M. Heideman:** Writing – review & editing. **J. Berkhof:** Writing – review & editing. **R.D.M. Steenbergen:** Writing – review & editing. **M.C.G. Bleeker:** Writing – review & editing, Supervision, Methodology, Conceptualization.

## Disclosure of interests

DAMH, and RDMS are minority stockholders of Self-screen BV, a spin-off company of Amsterdam UMC, location VUmc. Self-screen BV develops, manufactures and licenses the high-risk HPV assay and methylation marker assays for cervical cancer screening and holds patents on these tests.

## Funding

The authors received no financial support for this review.

## Declaration of competing interest

The authors declare the following financial interests/personal relationships which may be considered as potential competing interests: DAMH, and RDMS are minority stockholders of Self-screen BV, a spin-off company of Amsterdam UMC, location VUmc. Self-screen BV develops, manufactures and licenses the high-risk HPV assay and methylation marker assays for cervical cancer screening and holds patents on these tests.

## Data Availability

No data was used for the research described in the article.
